# Inhibition of Sphingosine Kinase-2 in a Murine Model of Lupus Nephritis

**DOI:** 10.1371/journal.pone.0053521

**Published:** 2013-01-03

**Authors:** Ashley J. Snider, Phillip Ruiz, Lina M. Obeid, Jim C. Oates

**Affiliations:** 1 Ralph H. Johnson Veterans Affairs Medical Center, Charleston, South Carolina, United States of America; 2 Division of Rheumatology and Immunology, Department of Medicine, Medical University of South Carolina, Charleston, South Carolina, United States of America; 3 Division of Immunopathology, Department of Pathology, University of Miami Miller School of Medicine, Miami, Florida, United States of America; 4 Department of Molecular and Cellular Biology and Pathobiology, Medical University of South Carolina, South Carolina, United States of America; University of Michigan, United States of America

## Abstract

Sphingosine-1-phosphate (S1P), a potent bioactive lipid, is emerging as a central mediator in inflammation and immune responses. We have previously implicated S1P and its synthetic enzyme sphingosine kinase (SK) in inflammatory and autoimmune disorders, including inflammatory bowel disease and rheumatoid arthritis. Generation of S1P requires phosphorylation of sphingosine by SK, of which there are two isoforms. Numerous studies have implicated SK1 in immune cell trafficking, inflammation and autoimmune disorders. In this study, we set out to determine the role of SK and S1P in lupus nephritis (LN). To this end, we examined S1P and dihydro-S1P (dh-S1P) levels in serum and kidney tissues from a mouse model of LN. Interestingly dh-S1P was significantly elevated in serum and kidney tissue from LN mice, which is more readily phosphorylated by SK2. Therefore, we employed the use of the specific SK2 inhibitor, ABC294640 in our murine model of LN. Treatment with ABC294640 did not improve vascular or interstitial pathology associated with LN. However, mice treated with the SK2 inhibitor did demonstrate decreases in glomerular pathology and accumulation of B and T cells in the spleen these were not statistically different from lpr mice treated with vehicle. LN mice treated with ABC294640 did not have improved urine thromboxane levels or urine proteinuria measurements. Both S1P and dh-S1P levels in circulation were significantly reduced with ABC294640 treatment; however, dh-S1P was actually elevated in kidneys from LN mice treated with ABC294640. Together these data demonstrate a role for SKs in LN; however, they suggest that inhibition of SK1 or perhaps both SK isoforms would better prevent elevations in S1P and dh-S1P and potentially better protect against LN.

## Introduction

SLE is a prototypic autoimmune disease that is characterized by humoral autoimmunity followed by cellular and innate immune responses. These responses in turn lead to inflammation and fibrosis in organs such as the kidney, skin, and joints. Lupus nephritis (LN) affects approximately 50% of patients with lupus, and five-year renal survival rates for patients with proliferative nephritis treated with cyclophosphamide vary from 95% among Caucasians to 58% among African-Americans [1]. Thus, more effective and less toxic therapies for LN are needed. Crescentic glomerular lesions are associated with poor prognosis in LN [2]. In addition, LN associated with podocyte damage leads to persistent proteinuria that can be refractory to therapy [3]. A novel therapeutic target for SLE is the activity of sphingosine kinase (SK).

Sphingolipids are bioactive lipids that are gaining increased attention due to their role in inflammation and cell survival. Sphingosine kinase (SK) exists in two isoforms, SK1 and SK2 and converts sphingosine to sphingosine-1-phosphate (S1P). This bioactive lipid is a known promoter of proliferation, migration, mitogenesis, and inflammation. SKs can also convert dihydro-sphingosine (dh-sphingosine) can also be converted to dh-S1P, typically by SK2 [4]. Cytokines such as tumor necrosis factor alpha (TNFα) and factors such as angiogenic growth factor, platelet derived growth factor (PDGF), and vascular endothelial growth factor (VEGF) all can act to transiently increase levels of S1P by activating SK to produce S1P. The majority of S1P actions are thought to involve S1P receptors 1–5 (S1PR 1–5), G protein-coupled receptors that are differentially expressed. These receptors can act through several different signaling pathways: S1PR1 and S1PR3 ultimately activate Rac to alter stress fiber function and promote cell migration toward S1P, and have been implicated in LN [5], [6]. S1PR1 has been demonstrated to play an essential role in the egress of lymphocytes from the lymph nodes into inflamed tissue and highlights the importance of S1P in immunity [7]. S1P also indirectly affects expression of cyclooxygenase-2 (COX2), the isoenzyme responsible for thromboxane (TXA_2_) production in LN [8]–[10].

Several studies suggest that SK activity may be a reasonable target in SLE. Pediatric patients with SLE, particularly those with lupus nephritis, have elevated serum levels of S1P [11]. In recent studies, a modulator of S1PR activity, FTY720, was effective in reducing proliferative glomerulonephritis in two murine models of LN [12], [13]. Development of this agent has been complicated by the development of bradycardia in human subjects and a lack of efficacy in preserving renal function in renal transplantation patients [14]. No published studies to date have determined if inhibiting the activity of SK is effective in delaying or reducing evidence of LN in a murine model of lupus.

This study was designed to determine the effect of preventative therapy with ABC294640, a competitive SK2 inhibitor, on the lupus phenotype in a murine model of lupus and to explore potential mechanisms through which this therapy might be acting to improve outcomes. MRL/MpJ-*Fas^lpr^*/2J (MRL/lpr) mice were treated with either ABC294640 or vehicle control and examined for standard lupus phenotype measures. MRL/lpr mice treated with ABC294640 exhibited reduction in clinical and pathological evidence of glomerulonephritis, but increased proteinuria in urine. The implications of these results for our understanding of sphingolipids and LN and possible roles for SK2 are examined.

## Methods

### Mice and General Protocol

All procedures were approved by the Ralph H. Johnson VA Medical Center Animal Care and Use Committee. MRL/MpJ-*Fas^lpr^*/2J (MRL/lpr) mice spontaneously develop vasculitis, inflammatory arthritis, and inflammatory glomerulonephritis in a manner similar to human lupus. These mice develop humoral autoimmunity at about twelve weeks, followed by clinical arthritis and nephritis [15]. Female MRL/lpr mice were purchased from Jackson Laboratory, housed in the Charleston VAMC Animal Facility, and monitored for common murine pathogens. Fifteen female (MRL/lpr) mice were divided into two groups: 1) ABC294640 (ABC, a sphingosine kinase inhibitor, 50 mg/kg twice daily by gavage, n = 15) or 2) vehicle (polyethylene glycol) by gavage twice daily (n = 15). MRL/MpJ (n = 10) mice were also gavaged twice daily with vehicle for non-disease comparison. Treatment began at 10 and proceeded to 20 weeks of age. In our experience, clinical disease activity in this model is greatest between 12 and 18 weeks, with half of mice dying of renal disease around 18–20 weeks of age. Urine was collected fortnightly from 10 to 20 weeks of age and analyzed for albumin and thromboxane B_2_ (TXB_2_, reflective of renal TXA_2_ production) levels. Total numbers of spleen cell subsets were determined by flow cytometry. Kidney tissues were collected at the time of sacrifice to characterize the lupus phenotype by light microscopy using a qualitative scoring system. Survival was compared between the groups. Mice were counted as non-survivors if found dead in cages or if they had developed hunched posture, ruffled fur, and had lost greater than 25% of their body weight. Mice fitting this latter characterization were sacrificed for humane reasons, and tissue was harvested for the above analyses. In our experience, mice in this condition do not survive more than 1–2 days.

### Treatment

Mice were treated with 50 mg/kg of ABC294640 (Apogee Biotechnology Corporation, Hummelstown, PA) or PEG vehicle alone by oral gavage twice daily. Repeated injections for 15 days and oral gavage up to 1000 mg/kg female Swiss-Webster mice did not show any overt toxicity or reductions in body weight. More detailed studies at 0, 100 or 250 mg per kg per day for 7 days showed no gross signs of toxicity or change in body weight of treated animals. Higher doses showed reductions in hematocrit of approximately 20% only in the 100 and 250 mg/kg/day dosing. There were no alterations noted in complete metabolic panel, coagulation parameters, or organ histopathology findings [16]. The 50 mg/kg dosing of this drug was chosen because this dose has been effective in treating murine models of inflammatory bowel disease and collagen-induced and adjuvant-induced arthritis [17], [18].

### Urine Analysis for Albumin

Urine was collected from individual mice house in metabolic cages for 24-hours. Urine was collected in an antibiotic solution, centrifuged to remove sediment, and stored at −80°C. Standard enzyme-linked immunosorbent assay techniques were used to detect urine albumin using the published methods of Sekine et al. and reported as milligrams of urine albumin per mouse per day [19].

### Sphingolipid Measurements

Serum, whole blood and kidney tissues were collected from mice at the time of sacrifice. Kidney tissue was homogenized and normalized to total protein content, while serum and whole blood were normalized by volume. Sphingolipid content was measured as previously described by the Lipidomics Shared Resource at the Medical University of South Carolina [20].

### Histological Grading of Renal Tissue

Renal tissue removed at the time of sacrifice was fixed in 4% buffered formaldehyde, embedded in paraffin, sectioned, and stained with hematoxylin and eosin by the MUSC core histopathology laboratory as described [21]. All histological grading was performed (by pathologist P.R.) using a scoring system described previously [22]. Briefly, glomeruli were graded for nine glomerular, nine interstitial, and one vascular elements, each on a zero to four scale with scores being additive. Glomerular elements were hypercellularity, hyperlobularity, crescents, mesangial expansion, necrosis, fibrosis, epithelial reactivity, vasculitis, thrombi, and thickened membranes. Scores for crescents and necrosis were doubled. Joints were scored similarly for synovial thickening, subsynovial fibrosis and fibroblasts, and subsynovial inflammation. Skin pathology was described by the extent of inflammation and vasculitis, including the presence of pigmented macrophages [22].

### Spleen Cell Analysis for Changes in Cell Subsets

Spleen cells were prepared for four color flow cytometry on a FACSCalibur flow cytometer by disruption of spleens and red cell lysis. A total of 10^6^ cells were incubated with anti-mouse CD16/CD32 for blocking Fc receptors. Cells were then incubated with primary Abs in PBS-BSA for 30 min at 4°C (BD Pharmingen, San Diego, CA), washed, and resuspended in 50 µl of PBS-BSA. The following antibodies were used to characterize spleen cell subsets as described [23]: total B cells (B220^+^, CD3^–^); total T cells (CD3^+^) as described [24].

### Urine TXB_2_ Analysis

Urine TXB_2_ levels were determined by competitive ELISA after immunoaffinity purification according to the manufacturer’s instructions (TXB_2_ Express, Cayman Chemical Company, Inc., Ann Arbor, MI). Urine dilution up to 1∶1000 prior to analysis was necessary to remove interference from the urine matrix.

### Immunohistochemical Staining of Renal Tissue for IgG and C3

Kidney tissue removed at the time of sacrifice was snap frozen in liquid nitrogen, fixed in acetone, washed in phosphate-buffered saline (PBS), incubated in 3% hydrogen peroxide for 10 minutes and rinsed in PBS. Slides were incubated in fluorescein isothiocyanate-conjugated monoclonal IgG antibody to C3 or IgG (Cappel; MP Biomedicals, Solon, OH) at a 1∶50 dilution in PBS for 1 hour in the dark at room temperature. Slides were then rinsed in PBS and distilled water and mounted with a cover slip using Vectashield hard set medium (Vector H-1400, Vector Laboratories, Burlingame, CA). Slides were scored (0–4) in a masked fashion by an experienced renal pathologist (S.E.S.) for the intensity and coverage of immunofluorescence in the glomerulus. Intensities were scored separately for basement membrane and mesangial deposits.

### Serum Anti-dsDNA and Immunoglobulin Isotype ELISAs

Blood was collected from mice at the time of euthanasia, and serum was separated from the clot. Serum anti-dsDNA antibody levels were determined by ELISA using DNA isolated from calf thymus as described [25].

### Statistical Analysis

Results were reported as the mean ± SEM in figures and as mean ± SD in supplemental tables. Differences among groups were determined by one-way ANOVA for normally distributed data, or by Mann Whitney or Wilcoxon Signed Rank Test for data not normally distributed. Missing data for the albumin analysis (due to death of mice) were carried forward from the last time point of collection.

## Results

### Mice with LN Demonstrate Increased Levels of Sphingosine Kinase Metabolites

**Figure 1:**
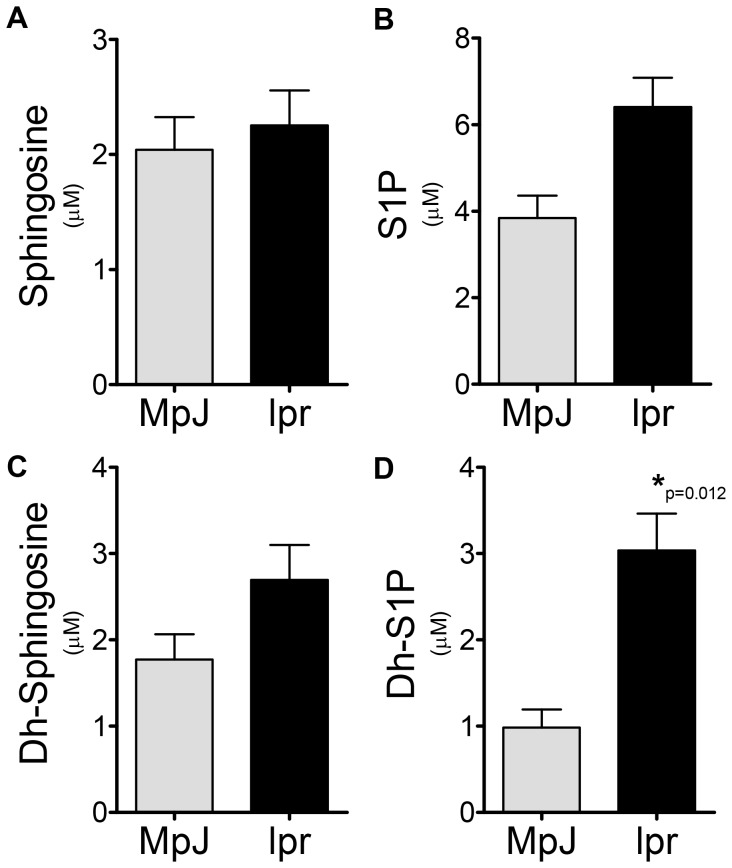
Lupus mice have elevated Dh-S1P levels in circulation. Serum was collected from MRL/lpr and MRL/MpJ mice and analyzed for sphingolipid content by the Lipidomics Shared Resource at MUSC, **A**) sphingosine, **B**) S1P, **C**) Dh-sphingosine and **D**) Dh-S1P. Data represent mean ± SEM, n = 6.

**Figure 2:**
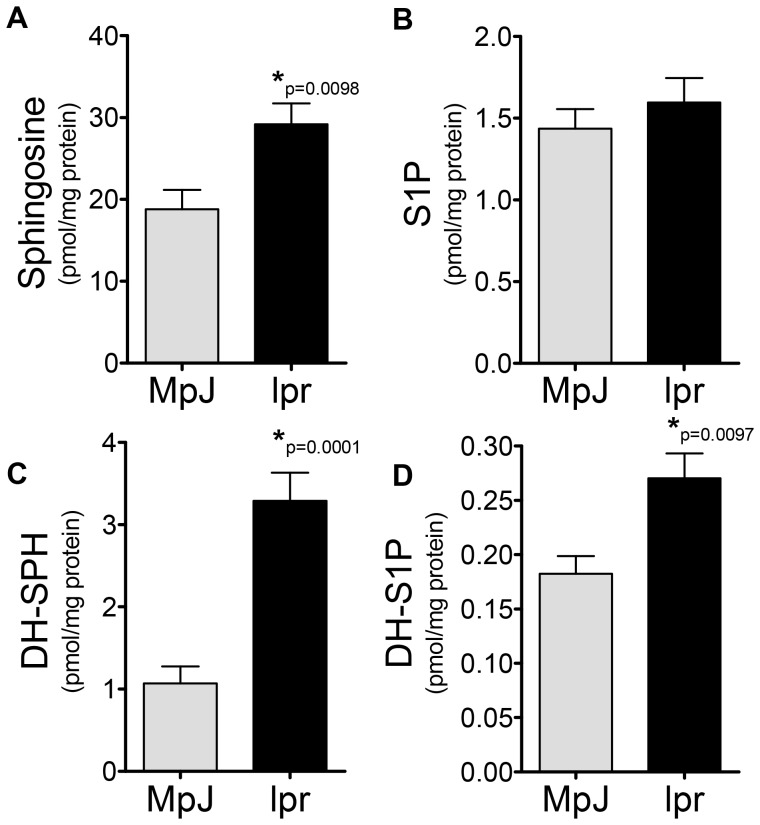
Sphingoid bases are elevated in kidney tissue from lupus mice. At 20 weeks of age, kidney tissue was collected from MRL/lpr and MRL/MpJ mice, homogenized and analyzed for sphingolipid content by the Lipidomics Shared Resource at MUSC, **A**) sphingosine, **B**) S1P, **C**) Dh-sphingosine and **D**) Dh-S1P. Data represent mean ± SEM, n = 6.

To determine if sphingosine kinase (SK) might be a rational target in this model of LN, sphingolipid profiles were determined in serum and cortical tissue from MRL/lpr disease and MRL/MpJ mice control mice at 20 weeks of age. It has previously been demonstrated that this is the age at which MRL/lpr mice have active nephritis. Serum ceramide species were generally not different; C16 and C18∶1 ceramides were slightly higher in the MRL/MpJ, while C24 ceramide was slightly higher in MRL/lpr mice. No serum ceramide species were statistically significant between MRL/MpJ and MRL/lpr mice (data not shown). However, serum levels of SK metabolites were increased in MRL/lpr mice (Figure 1B and D), significantly so for dh-S1P (Figure 1B). Levels of neither sphingosine (SPH) nor dh-sphingosine (dh-SPH) were significantly altered in serum from MRL/lpr mice when compared to MRL/MpJ (Figure 1A and C). When kidney sphingolipid levels were examined SPH, dh-SPH and dh-S1P were significantly elevated in MRL/lpr mice (Figure 2A, C and D), while no significant changes were observed in S1P levels. These data suggest that SK activity is increased in the kidney of MRL/lpr mice, either by direct activation or by increases in substrate availability, and that perhaps SK2, which has a higher affinity for dh-sphingosine, may be a rational target for therapy.

### ABC294640 has No Effect on Glomerular Pathology

**Figure 3:**
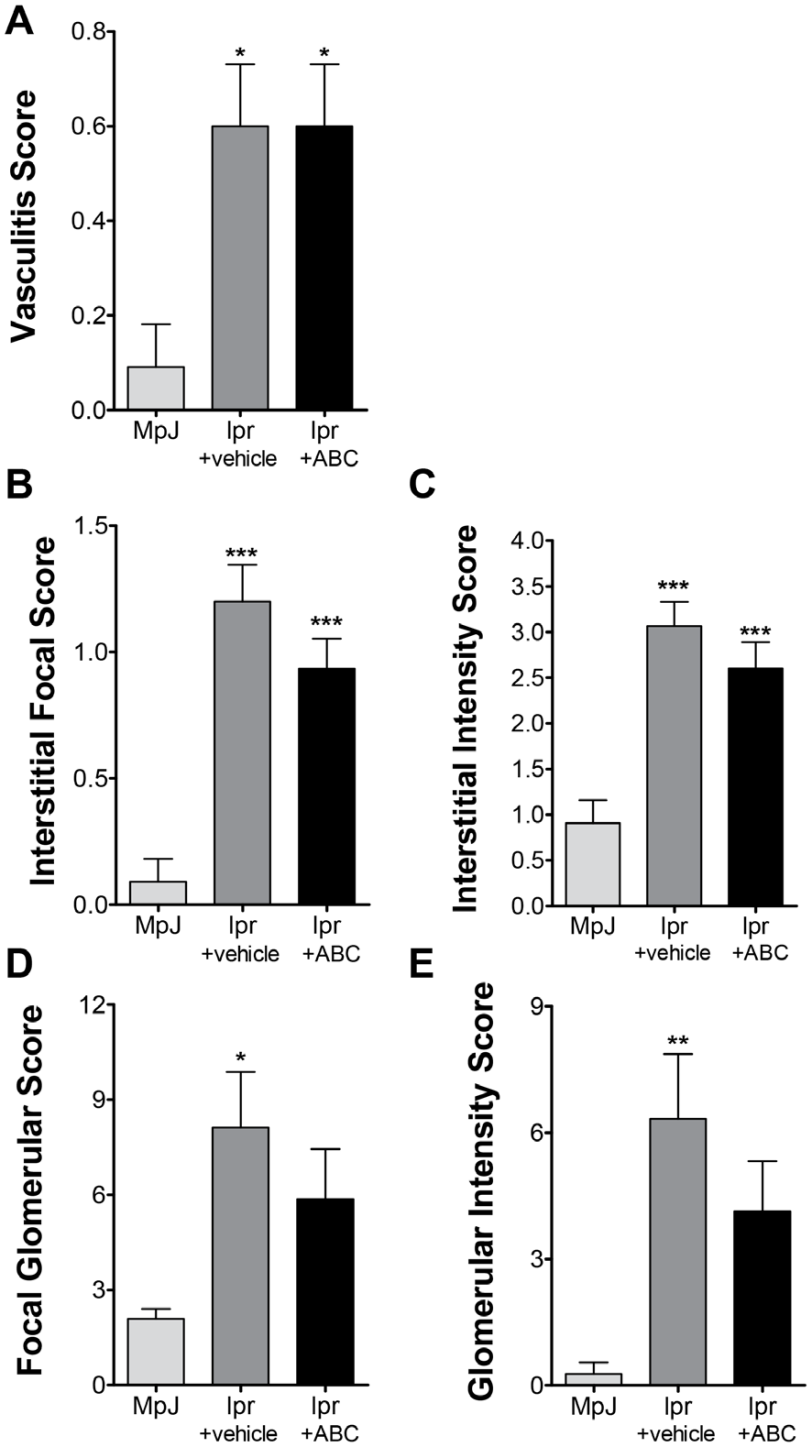
Glomerular pathology is not altered by treatment with ABC294640. MRL/MpJ mice were treated with vehicle and MLR/lpr mice were treated with vehicle or ABC294640 for 10 weeks. Kidneys were collected and sectioned for H&E staining. A blinded pathologist scored kidneys for **A**) vasculitis, interstitial **B**) focal or **C**) intensity, and glomerular **D**) focal or **D**) **i**ntensity scores. Data represent mean ± SEM, n≥10; *p<0.05, **p<0.01, ***p<0.001 treated vs. MPJ.

After observing increases in dh-sphingosine and dh-S1P in kidney tissue from MRL/lpr mice, we set out to determine if inhibition of SK2 with ABC294640, a specific SK2 inhibitor [16], would improve pathobiology associated with LN. MRL/MpJ mice were treated with vehicle as a biologic control strain, and MRL/lpr mice were treated with either vehicle (lpr+vehicle) or ABC294640 (lpr+ABC) 50 mg/kg via oral gavage twice daily between 10 and 20 weeks of age. Kidney tissue was collected and examined for pathology as described in the Methods, by a blinded pathologist. ABC294640 had no significant effect on either the vasculitis or interstitial pathology scores (Figure 3A, B and C). MRL/lpr mice treated with vehicle had significant increases in glomerular pathology (Figure 3D and E) as expected. Lpr mice treated with ABC294640 did not have significantly different glomerular pathology scores from MpJ mice treated with vehicle or from lpr+vehicle mice (Table S1). These data demonstrate that mice treated with the SK2 inhibitor demonstrate a trend towards decreased glomerular disease in LN but does not prevent vasculitis or interstitial pathology associated with LN.

### ABC294640 does not Effect B and T Cell Counts in the Spleen of Mice with LN

**Figure 4:**
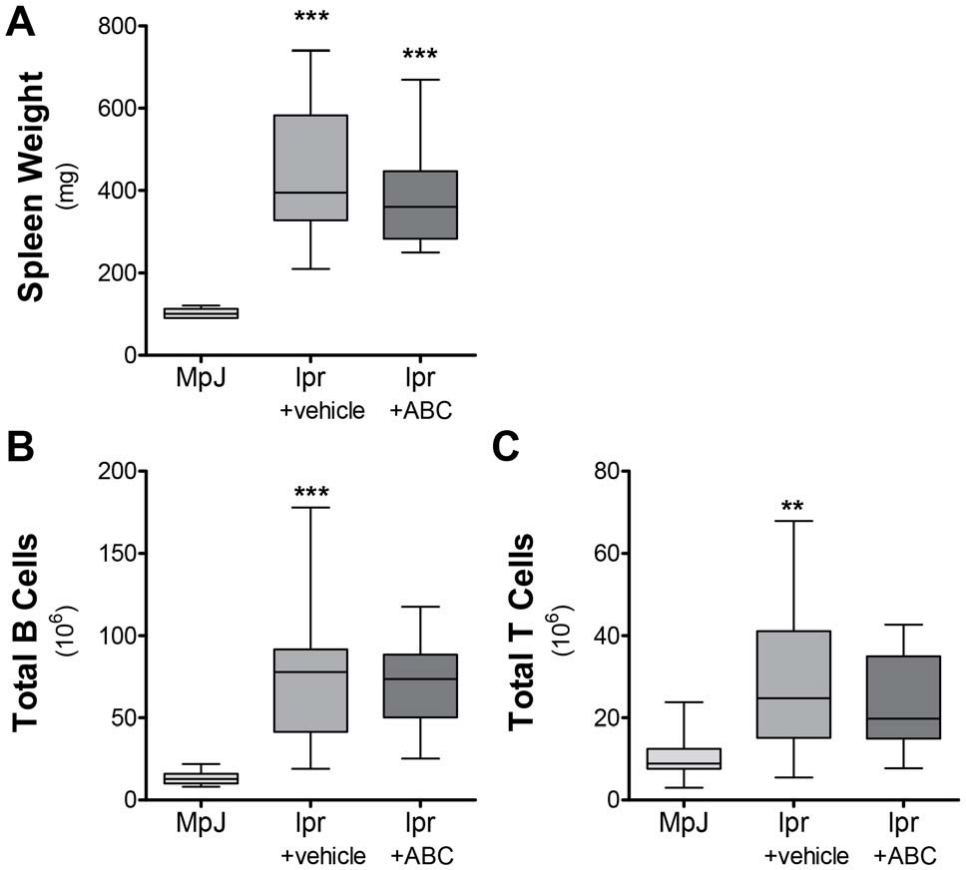
Spleen weight and lymphocyte counts are not significantly altered by ABC294640. MRL/MpJ mice were treated with vehicle and MLR/lpr mice were treated with vehicle or ABC294640 for 10 weeks. **A**) Spleen weight was measured following euthanasia from mice at 20 weeks of age. **B**) Total B and **C**) T cell populations were analyzed using FACs flow cytometry. Data represent mean ± SEM, n≥10; **p<0.01, ***p<0.001 treated vs. MPJ.

Given that S1P is important in homing of lymphocytes and decreases in S1P levels have been associated with increases in B and T cells in the spleen [26], we explored whether spleen cell subsets were changed by ABC294640 therapy on the presumption that increased homing to sites of inflammation would result in lower spleen counts. Spleen cells were analyzed for CD3+ T cell subsets (CD4, Cd8, total, newly activated, and memory) and B cell (germinal center, marginal zone, follicular, newly activated, Class B1 and B2) subsets and NK cell, macrophage, and granulocyte populations. Only the total T-cell and B-cell populations were altered in MRL/lpr mice treated with ABC294640. There were significant increases in spleen weight in lpr+vehicle and lpr+ABC when compared to MpJ+vehicle (Figure 4A). Total splenic B and T cells increased in lpr+vehicle mice and when compared to MpJ+vehicle (Figure 4B and C), but were not stastistcally different from lpr+ABC cell counts (Table S2). These results demonstrate that increases in circulating S1P could play a role in egress of B and T cells in the spleen, but this is not completely controlled by SK2.

### Urine Proteinuria is not Decreased in LN by ABC294640

**Figure 5:**
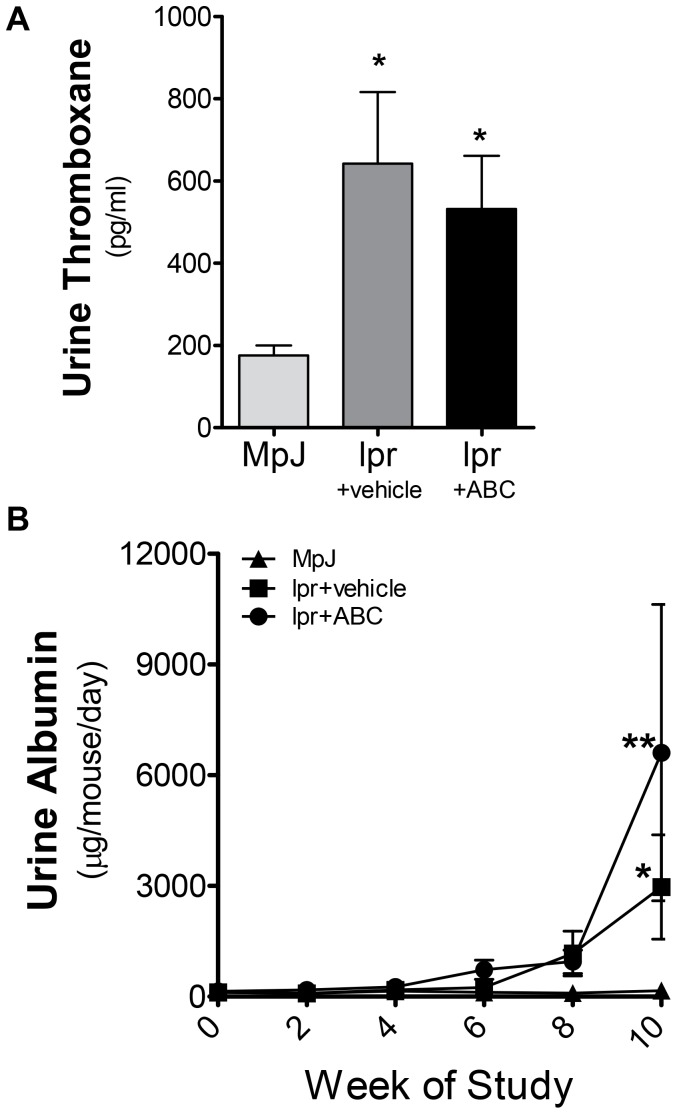
ABC294640 does not prevent increases in urine thromboxane or albumin levels. After 10 weeks of ABC294640 or vehicle administration, 24-hour urine samples were collected from MRL/MpJ and MRL/lpr mice. **A**) Urine thromboxane and **B**) urine albumin levels were measured by ELISA. Data represent mean ± SEM, n≥10; *p<0.05 treated vs. MPJ.

To determine if the slight improvement in pathology was associated with decreased urine thromboxane and/or urine albumin levels, 24-hour urine collections were performed every 2 weeks, from ages 10–20 weeks in all mice for albumin levels and thromboxane measured at the conclusion of the study. MRL/lpr mice treated with both vehicle and ABC294640 exhibited significant increases in thromboxane levels at 20 weeks of age (Figure 5A). Additionally both MRL/lpr mice treated with vehicle and ABC294640 exhibited a significant increase in urine albumin levels over MRL/MpJ mice (Figure 5B); however, treatment with ABC294640 did not significantly reduce alter proteinuria when compared to MRL/lpr mice treated with vehicle (Table S3).

#### ABC294640 significantly increases kidney dh-S1P levels in kidneys from mice with LN

**Figure 6:**
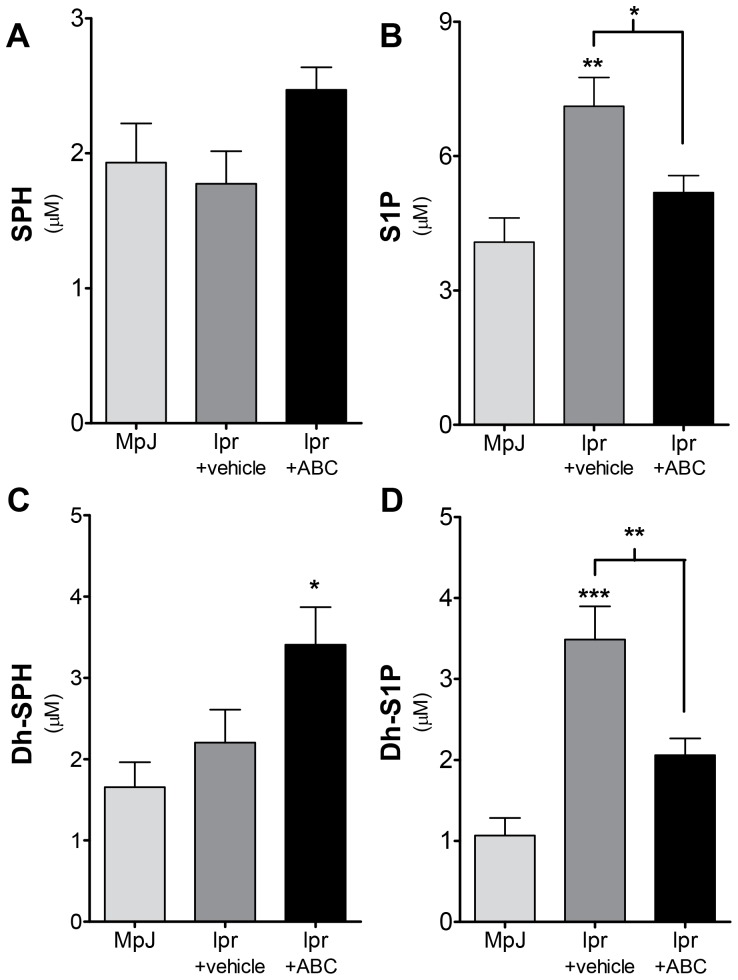
ABC294640 prevents accumulation of Dh-S1P and S1P in circulation of lupus mice. After 10 weeks of ABC294640 or vehicle administration, whole blood was collected from MRL/MpJ and MRL/lpr mice. Blood was analyzed for sphingolipid content by the Lipidomics Shared Resource at MUSC, **A**) sphingosine, **B**) S1P, **C**) Dh-sphingosine and **D**) Dh-S1P. Data represent mean ±SEM, n≥10; *p<0.05, **p<0.01, ***p<0.001 treated vs. MPJ, unless otherwise indicated.

**Figure 7:**
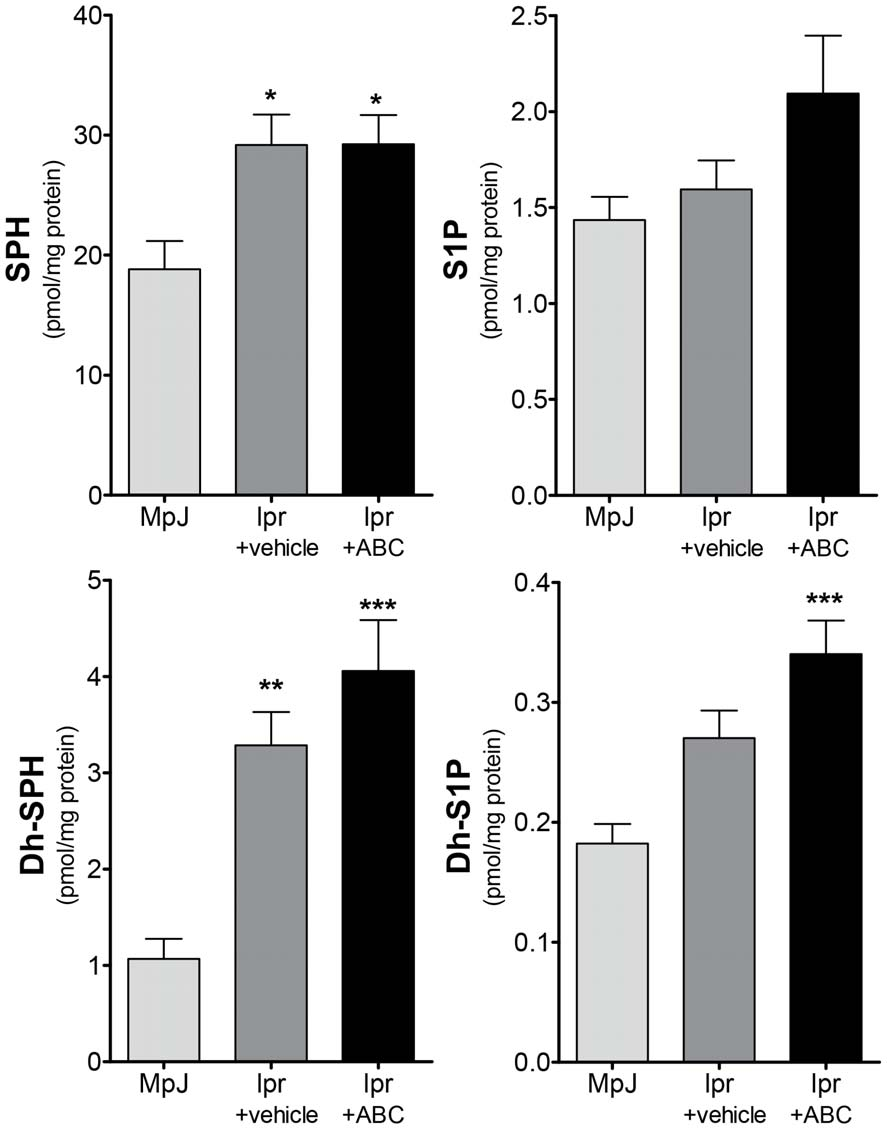
Increase in kidney Dh-S1P is not prevented by ABC294640 in lupus mice. After 10 weeks of ABC294640 or vehicle administration, kidneys were collected from MRL/MpJ or MLR/lpr mice. Kidney tissue was homogenized and analyzed for sphingolipid content by the Lipidomics Shared Resource at MUSC, **A**) sphingosine, **B**) S1P, **C**) Dh-sphingosine and **D**) Dh-S1P. Data represent mean ± SEM, n≥10; *p<0.05, **p<0.01, ***p<0.001 treated vs. MPJ.

After observing a mixed result for the use of ABC294640 in prevention of LN, we examined sphingolipid changes in both circulation and kidney tissues from from lpr+vehicle and lpr+ABC mice. Dh-sphingosine was increased in circulation in mice receiving ABC294640 when compared to MRL/MpJ mice (Figure 6C), while the whole blood increase in both S1P and dh-S1P associated with LN was prevented by ABC294640 (Figure 6B and D). These data suggest that ABC294640 alters circulating sphingolipid levels and that this may effect lymphocyte trafficking. However, this did not explain the obvious alteration of renal function observed with ABC294640 in LN. Therefore, we examined sphingolipid levels in kidney tissues. Sphingosine and dh-SPH were significantly increased in MRL/lpr mice treated with both vehicle and ABC294640 (Figure 7A&C). However, only MRL/lpr mice treated with ABC294640 exhibited a significant increase in dh-S1P levels (Figure 7D and Table S4). These data suggest that dh-S1P synthesis in the kidney in this model is not mediated by the target of ABC294640 therapy, SK2 and is likely due to SK1 activity.

## Discussion

This is the first study to target SK activity in LN. We set out to determine if a specific isoform of SK was involved in LN. We observed increases in dh-S1P in both circulation and kidney tissue from MRL/lpr mice and therefore examined inhibition of SK2 in a murine model of LN, as SK2 has a higher affinity for dh-S1P than does SK1 [4]. To this end we utilized an inhibitor for SK2, ABC294640 in MRL/lpr mice. Our data demonstrate that treatment with ABC294640 in the MRL/lpr mouse model does decrease some aspects of renal pathology associated with LN but does not improve albuminuria as one marker of glomerular filtration barrier integrity. The observed improvement in glomerular pathology is not likely due to significant reductions in anti-dsDNA antibody levels or IgG deposition, as these outcomes did not change significantly with therapy (Figure S1). Therefore, the effect of therapy was likely downstream of immune complex deposition. Treatment with ABC294640 decreased circulating S1P and dh-S1P levels, but kidney levels of dh-S1P were significantly elevated in MRL/lpr mice treated with the SK2 inhibitor. The data reported here suggest that targeting SK2 activity in this model of murine LN reduced progression of glomerular disease; however, targeting SK1 may prevent the accumulation of dh-S1P in kidney tissue and improve kidney pathology, albuminuria, and function.

In human lupus, circulating S1P levels are increased [11], suggesting that one or both isoenzymes of SK have increased activity in lupus. Sphingolipids have been previously implicated in animal models of LN, however, these studies have focused more on the function of S1P receptors (S1PRs) in lymphocyte trafficking and the use of FTY720 (a S1PR superagonist that engages and induces internalization of most S1PRs). In the New Zealand Black x New Zealand White (NZBxNZW)_F1_ model of lupus, FTY720 reduced glomerular inflammation as in our study [13]. In another LN model, BXSB, FTY720 prevented proteinuria as well as increased survival. (Of note here, there were no significant differences in survival between vehicle and ABC294640 groups, as the study was conducted only until 20 weeks to examine pathobiological changes with ABC294640 as opposed to effect on survival – data not shown). FTY720 treatment also exhibited decreased mesangial cell proliferation and inflammatory cell infiltration into the kidney [6]. Of note, for FTY720 to be activated and act on S1PRs, it must first be phosphorylated by SK2, suggesting that SK2 is active in lupus. However, our studies indicate the inhibition of active SK2 is insufficient to significantly improve pathologies associated with LN. In another study examining the modulation of S1PRs, KRP203 treatment resulted in increased survival and decreased T cell infiltration into the kidney of MRL/lpr mice [5]. These studies clearly indicate a role for S1PR in modulating immune cell infiltration in an animal model of LN.

Along with the results in the literature demonstrating modulation of S1PRs and immune cell trafficking, our current study suggests that S1P and dh-S1P are potential pathogenic mediator in LN. Several mechanisms for this effect are possible but are not directly addressed by this study. First, there was a trend toward a reduction in urine TXB_2_, with ABC294640 treatment; however, not a significant reduction. This corroborates a large field of literature, demonstrating that SK1 derived S1P induces the expression of cyclooxygenase-2 (COX2). In humans and in murine models of proliferative nephritis, COX2-depenent renal TXA_2_ production is increased [10], [27], [28]. This further indicates that inhibition of SK1 may prove to be a more beneficial therapeutic in LN. Second, S1P, through S1PR1/3, induces cells to migrate from a low concentration of S1P in lymph tissue to a higher concentration in inflamed tissue [29]. SK1 is highly expressed in mesangial cells, making mesangial cells a potential resident cell source of S1P in the glomerulus [30]. Third, S1P may induce resident or infiltrating cells to produce chemokines that in turn induce infiltration of inflammatory cells. In macrophages, S1PRs 1/3 signal primarily through the G protein Gi to ultimately induce IL8 and MCP1 expression. Taken together, this suggests that inhibition of SK1 or both SKs may prove better targets in LN.

Complete response to therapy for LN is the exception rather than the rule, and progression to end-stage renal disease occurs at alarming rates with the current standard of care [1]. Glomerular lesions that portend a poor prognosis include chronic, crescentic and segmental lesions [2]. Treatment refractory proteinuria occurs frequently in LN and is associated with podocyte damage [3]. This study demonstrates that ABC294640 therapy improves glomerular lesions, but does not prevent proteinuria in MRL/lpr mice. Because S1P and dh-S1P levels are increased in renal tissue in MRL/lpr mice and these levels are not increased by ABC294640, SK1, and not SK2, may be the active isotype and thus a more effective target in LN.

## Supporting Information

Figure S1 **ABC294640 does not significantly alter IgG, C3 or dsDNA in LN mice.** After 10 weeks of ABC294640 or vehicle administration, kidneys were collected from MRL/MpJ or MLR/lpr mice, **A) & B)** IgG and **C)** C3 were examined by immunohistochemistry and quantified. Serum was collected at the time of euthanasia and **D)** dsDNA measured using Elisa. Data represent mean ± SEM, n≥10; *p<0.05 treated vs. MPJ.
(TIF)

Table S1 **Pathology Scores.** Following 10 weeks of treatment with either vehicle or ABC294640 kidney pathology was assessed by a blinded pathologist Values are mean ± SD. *Significantly different from MpJ+vehicle, *p<*0.05; ***Significantly different from MpJ+vehicle, p<0.01 by One-way ANOVA; n≥10.
(PDF)

Table S2 **Spleen Weight, splenic T and B cell counts.** Following 10 weeks on either vehicle or ABC294640 spleens were weighted and assessed for B and T cell counts. Values are mean ± SD. **Significantly different from MpJ+vehicle, *p<*0.01; ***Significantly different from MpJ+vehicle, p<0.001 by One-way ANOVA; n≥10.
(PDF)

Table S3 **Urine albumin measurements.** Urine samples were collected from mice every two weeks of the study, beginning at week 0. Values are mean ± SD. ^†^Significantly different from lpr+vehicle, *p*<0.05; *Significantly different from MpJ+vehicle, *p*<0.05; **Significantly different from MpJ+vehicle *p*<0.01 by One-way ANOVA; n≥10.
(PDF)

Table S4 **Kidney Sphingolipid Measurements.** Sphingolipid levels were analyzed by ESI/MS/MS from kidney homogenate following 10 weeks of either treatment with vehicle or ABC294640. Values are mean ± SD. *Significantly different from MpJ+vehicle, *p*<0.05; **Significantly different from MpJ+vehicle; *p*<0.01, ***Significantly different from MpJ+vehicle, p<0.001 by One-way ANOVA; n≥10.
(PDF)
