# Supplementary figures and images for: Inhibition of Sphingosine Kinase-2 in a Murine Model of Lupus Nephritis
Source: PLoS One. 2013 Jan 3;8(1):e53521. doi: 10.1371/journal.pone.0053521 (PMC3536755; doi:10.1371/journal.pone.0053521)

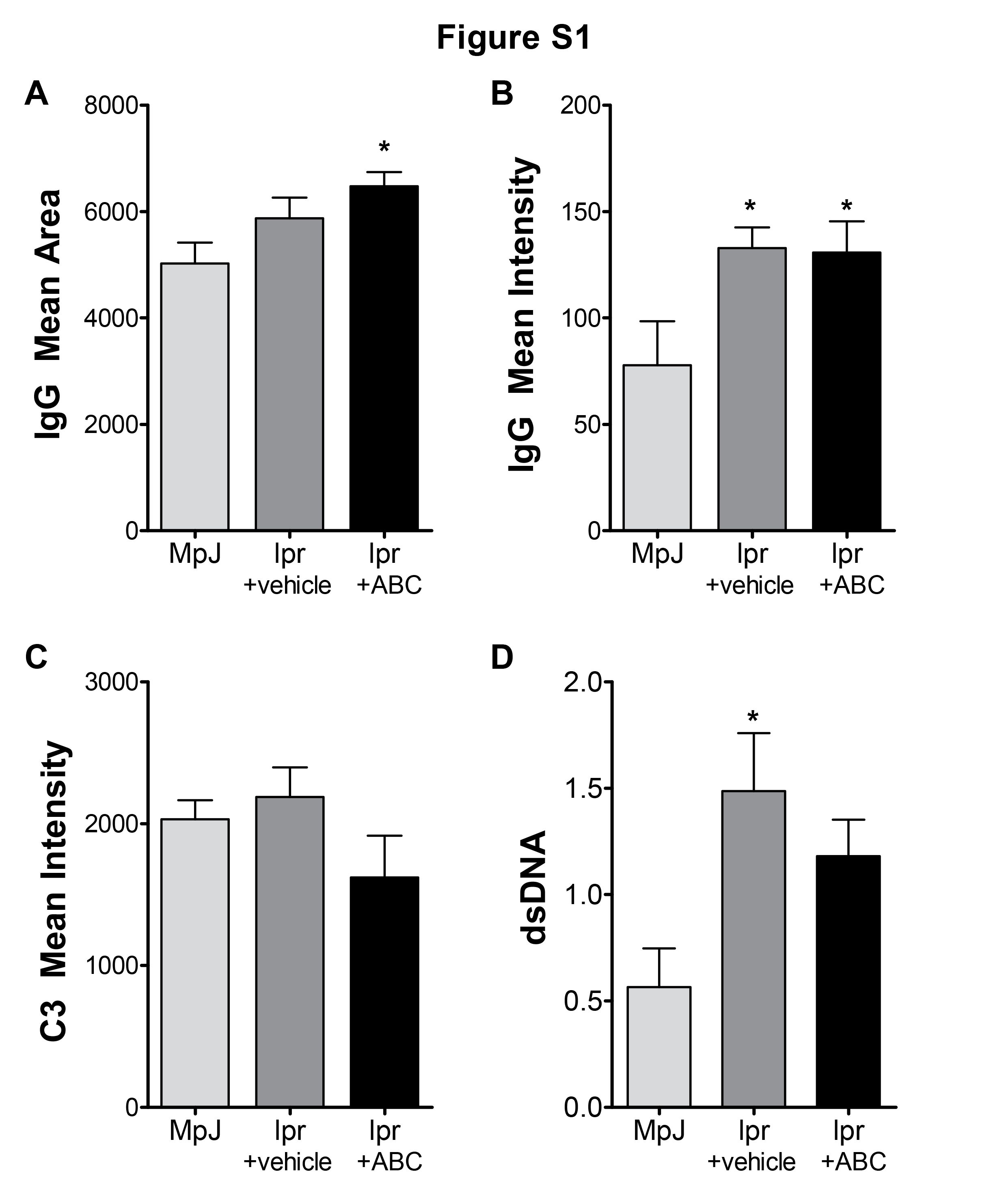

Supplement: Figure S1 — ABC294640 does not significantly alter IgG, C3 or dsDNA in LN mice. After 10 weeks of ABC294640 or vehicle administration, kidneys were collected from MRL/MpJ or MLR/lpr mice, A) & B) IgG and C) C3 were examined by immunohistochemistry and quantified. Serum was collected at the time of euthanasia and D) dsDNA measured using Elisa. Data represent mean ± SEM, n≥10; *p<0.05 treated vs. MPJ. (TIF) [file pone.0053521.s001.tif]
