# Supplementary material for: Inhibition of Sphingosine Kinase-2 in a Murine Model of Lupus Nephritis
Source: PLoS One. 2013 Jan 3;8(1):e53521. doi: 10.1371/journal.pone.0053521 (PMC3536755; doi:10.1371/journal.pone.0053521)
Supplement: Table S1 — Pathology Scores. Following 10 weeks of treatment with either vehicle or ABC294640 kidney pathology was assessed by a blinded pathologist Values are mean ± SD. *Significantly different from MpJ+vehicle, p<0.05; ***Significantly different from MpJ+vehicle, p<0.01 by One-way ANOVA; n≥10. (PDF) [file pone.0053521.s002.pdf]

Table S1

Treatment Group	Vasculitis Score	Interstitial Score		Glomerular Score	
		Focal	Intensity	Focal	Intensity
MpJ + vehicle	0.00 ± 0.00	0.00 ± 0.00	0.70 ± 0.48	1.80 ± 0.42	0.00 ± 0.00
lpr + vehicle	0.67 ± 0.49**	3.07 ± 1.03***	1.20 ± 0.56***	8.20 ± 6.73***	6.33 ± 5.94***
lpr + ABC294640	0.60 ± 0.51*	1.00 ± 0.38***	2.80 ± 0.86***	6.13 ± 5.94*	4.33 ± 4.48*
