# Supplementary material for: Inhibition of Sphingosine Kinase-2 in a Murine Model of Lupus Nephritis
Source: PLoS One. 2013 Jan 3;8(1):e53521. doi: 10.1371/journal.pone.0053521 (PMC3536755; doi:10.1371/journal.pone.0053521)
Supplement: Table S2 — Spleen Weight, splenic T and B cell counts. Following 10 weeks on either vehicle or ABC294640 spleens were weighted and assessed for B and T cell counts. Values are mean ± SD. **Significantly different from MpJ+vehicle, p<0.01; ***Significantly different from MpJ+vehicle, p<0.001 by One-way ANOVA; n≥10. (PDF) [file pone.0053521.s003.pdf]

Table S2

Treatment Group	Spleen Weight (mg)	Total B Cells ($1x10^6$)	Total T Cells ($1x10^6$)
MpJ + vehicle	101.00 ± 11.97	13.35 ± 4.00	10.54 ± 5.56
lpr + vehicle	431.43 ± 163.98***	95.17 ± 79.05***	29.82 ± 17.90**
lpr + ABC294640	378.57 ± 117.00***	73.07 ± 25.17***	22.6 ±11.01
