# Supplementary material for: Inhibition of Sphingosine Kinase-2 in a Murine Model of Lupus Nephritis
Source: PLoS One. 2013 Jan 3;8(1):e53521. doi: 10.1371/journal.pone.0053521 (PMC3536755; doi:10.1371/journal.pone.0053521)
Supplement: Table S3 — Urine albumin measurements. Urine samples were collected from mice every two weeks of the study, beginning at week 0. Values are mean ± SD. †Significantly different from lpr+vehicle, p<0.05; *Significantly different from MpJ+vehicle, p<0.05; **Significantly different from MpJ+vehicle p<0.01 by One-way ANOVA; n≥10. (PDF) [file pone.0053521.s004.pdf]

Table S3

**Treatment Group**	**Week of Study**	**Albuminuria** (ug/mouse/day)
MpJ + vehicle	0	115.42 ± 71.24
lpr + vehicle	0	118.66 ± 112.26
lpr + ABC294640	0	145.21 ± 139.92
MpJ + vehicle	2	87.50 ± 21.50
lpr + vehicle	2	88.91 ± 70.91
lpr + ABC294640	2	182.56 ± 208.30[†]
MpJ + vehicle	4	148.86 ± 95.95
lpr + vehicle	4	186.38 ± 136.23
lpr + ABC294640	4	265.39 ± 272.31
MpJ + vehicle	6	114.99 ± 64.33
lpr + vehicle	6	249.36 ± 159.75
lpr + ABC294640	6	730.41 ± 1013.61*
MpJ + vehicle	8	94.63 ± 52.70
lpr + vehicle	8	1169.39 ± 2348.34
lpr + ABC294640	8	947.40 ± 1221.51**
MpJ + vehicle	10	162.08 ± 104.63
lpr + vehicle	10	2967.54 ± 5477.15*
lpr + ABC294640	10	6613.22 ± 15548.67**
