# Supplementary material for: Inhibition of Sphingosine Kinase-2 in a Murine Model of Lupus Nephritis
Source: PLoS One. 2013 Jan 3;8(1):e53521. doi: 10.1371/journal.pone.0053521 (PMC3536755; doi:10.1371/journal.pone.0053521)
Supplement: Table S4 — Kidney Sphingolipid Measurements. Sphingolipid levels were analyzed by ESI/MS/MS from kidney homogenate following 10 weeks of either treatment with vehicle or ABC294640. Values are mean ± SD. *Significantly different from MpJ+vehicle, p<0.05; **Significantly different from MpJ+vehicle; p<0.01, ***Significantly different from MpJ+vehicle, p<0.001 by One-way ANOVA; n≥10. (PDF) [file pone.0053521.s005.pdf]

Table S4

Treatment Group	Total Ceramide	SPH	S1P	DH-SPH	DH-S1P
MpJ + vehicle	699.43 ± 145.05	18.82 ± 7.45	1.44 ± 0.38	1.07 ± 0.66	0.18 ± 0.05
lpr + vehicle	778.35 ± 155.39	29.17 ± 9.89*	1.60 ± 0.58	3.29 ± 1.34**	0.27 ± 0.09
lpr + ABC294640	756.86 ± 304.30	29.25 ± 9.44*	2.09 ± 1.17	4.06 ± 2.05***	0.34 ± 0.11***
